# Significance of Albuminuria

**Published:** 1882-07

**Authors:** 


### SIGNIFICANCE OF ALBUMINURIA.

At the meeting of the Cambridge Medical Society on April 14, Mr. Carter introduced the subject of the significance of albuminuria when not dependent on Bright's disease. He remarked on the frequent difficulty of satisfactorily ascertaining the precise meaning of albuminuria when unattended by kidney disease. Various albuminoid substances were known to occur in the urine, and besides the several forms of albumen and the globulins, there also sometimes occurred the several ferments, ptyalin, pepsin, and trypsin. The results of various observers went to prove that out of a given number of cases of albuminuria only about half would be of renal origin, and the remainder would be found to arise from other conditions. In one class of cases it was to be referred to the nervous system—neurotic albuminuria—and, like diabetes, it was not infrequently found to follow prolonged mental anxiety. The form in which it occurred in young men, the so-called albuminuria of adolescents, had been regarded as neurotic, but Mr. Carter thought it more probable that it was connected with the sexual function; were it of nervous origin it would occur in young women and girls, which it was seldom known to do. It would seem to depend upon some condition peculiar to males. Albuminuria frequently signified mal-assimilation of the albuminoid elements of the food. If the amyloids of the food failed to be normally assimilated, he suggested that a set of conditions occurred of which diabetes was the type; if the assimilation of the albuminoids were faulty, that other conditions arose which were indicated by albuminuria. The late Dr. Parks had called attention to the condition here referred to, which he named "food albuminuria." In yet another class of cases albuminuria signified some interference with the circulation of the blood, or some abnormal condition of the blood itself. In those affections of the liver in which it was a symptom, it might depend either on abnormal circulation, in which case serum albumen would appear, or on defective metamorphosis of the albuminoids, when it would almost certainly be due to the presence of some other albuminoid. In morbid pulmonary conditions also it might indicate either interrupted circulation or defective pulmonary excretion. When it occurred in pregnancy it appeared that impediment to the venous circulation was commonly the main factor, but there was also an altered quality of the blood itself. Dr. Bradbury remarked that the subject was one in which he had taken a special interest. With regard to the albuminuria of adolescents, he had in his practice been consulted by many undergraduates affected by it, and had, therefore, unusual opportunities for investigating its cause. In a large proportion of such cases the albumen was only to be found after breakfast, and he had come to the conclusion that it was often due to seminal fluid finding its way into the urine in connection with the act of defecation. There could be no doubt as to the existence of many varieties of albuminuria, but it was difficult to determine the different forms. Often it was an accompaniment of indigestion, and sometimes occurred only after the indigestion of certain articles of food, as in one case which had come under his own care, in which it occurred only after the patient had partaken of boiled beef. He had seen cases also depending on hepatic derangement due to alcohol or other excess, and had noted the disappearance of the albumen when the heptic enlargement subsided. Albuminuria with high arterial tension meant, as a rule, commencing Bright's disease; when accompanied by low tension the probabilities were in favor of there being no renal disease.—*Lancet*, May 13, 1882.

